# Association between flourishing mental health and occupational stress among workers of Tsukuba Science City, Japan: a cross-sectional study

**DOI:** 10.1186/s12199-019-0823-7

**Published:** 2019-11-27

**Authors:** Daisuke Hori, Yuichi Oi, Yuh Ohtaki, Christina-Sylvia Andrea, Tsukasa Takahashi, Nagisa Shiraki, Tomohiko Ikeda, Yu Ikeda, Shotaro Doki, Shinichiro Sasahara, Ichiyo Matsuzaki

**Affiliations:** 1 Faculty of Medicine, University of Tsukuba, 1-1-1 Tennodai, Tsukuba, Ibaraki, 305-8575 Japan; 2 Hospital Bando, 411 Kutsukake, Bando, Ibaraki, 306-0515 Japan; 3 Graduate School of Comprehensive Human Sciences, University of Tsukuba, 1-1-1 Tennodai, Tsukuba, Ibaraki, 305-8575 Japan; 4 International Institute for Integrative Sleep Medicine, University of Tsukuba, 1-1-1 Tennodai, Tsukuba, Ibaraki, 305-8575 Japan

**Keywords:** Flourishing, Japan, Mental health, Occupational stress, Workplace

## Abstract

**Background:**

Flourishing, defined as the coexistence of hedonic and eudaimonic well-being, is the most favorable end of the mental health spectrum. A growing body of evidence has demonstrated the association between flourishing mental health and favorable work-related outcomes. However, epidemiology of flourishing mental health is scarce in Japan. Moreover, the relationship between flourishing mental health and occupational stress has not been elaborated. Therefore, the aim of this study was to elucidate (1) the prevalence of flourishing mental health and (2) the association between flourishing mental health and occupational stress among Japanese workers.

**Methods:**

The present survey was conducted in from February to March 2017 via an anonymous, self-administered, and web-based questionnaire among workers in Tsukuba Science City, Japan. Mental Health Continuum Short Form was used to assess flourishing mental health. We performed binomial logistic regression analyses to calculate the adjusted odds ratios (AOR) and 95% confidence intervals (CI) of occupational stress for flourishing mental health, controlling for sociodemographic factors.

**Results:**

A total of 7012 respondents (4402 men, 2610 women) were analyzed. The overall prevalence of flourishing mental health among the respondents was 12.4%. Full-time (permanent) workers were less likely to be flourishing. Reward from work (AOR = 2.34, 95% CI = 2.04–2.68), support from colleagues and superiors (AOR = 1.67, 95% CI = 1.44–1.94), and workload (AOR = 1.19, 95% CI = 1.05–1.36) were positively associated with flourishing mental health, whereas mental workload (AOR = 0.65, 95% CI = 0.57–0.75) was inversely associated with flourishing mental health.

**Conclusions:**

The findings of the present study shed light on the association between flourishing mental health and occupational stress.

## Background

Mental health has long been understood as the absence of mental illness; however, this definition has changed considerably over the past several decades. In 2004, the World Health Organization defined mental health as “a state of well-being in which every individual realizes his or her own potential, can cope with the normal stresses of life, can work productively and fruitfully, and is able to make a contribution to her or his community” [1]. Since that time, the concept that “the absence of mental diseases does not imply the presence of full mental health” has become widely accepted. Previous studies have confirmed that the symptoms of mental health and mental illness do not reflect opposite ends of the same continuum, but rather, load onto two separate but related factors [2, 3]. This means that to maintain and enhance human well-being, it is important to focus on not only mental illness, but also the level of mental health. Diener et al. [4] reported that flourishing individuals are able to develop warm, trusting relationships with others and are willing to develop their potential to grow and expand as a person.

The most favorable end of the mental health spectrum is referred to as flourishing [5], which is defined as a combination of two conceptual traditions of mental well-being: hedonic and eudaimonic well-being [6]. The hedonic tradition focuses on emotional well-being through the measurement of satisfaction with life and positive affect [7], while the eudaimonic tradition focuses on psychological well-being [8] and social well-being [9], and assesses how well individuals see themselves functioning in life, such as social contributions, social integration, autonomy, and personal growth. Measurement of flourishing is currently based on data collected via self-administered questionnaires. A number of epidemiological studies on flourishing mental health have been conducted worldwide. The prevalence of flourishing was estimated to range from 11.7 to 76.9% in general population [10–14]. Longitudinal evidence has indicated that flourishing mental health has favorable effects on individual health. A 10-year cohort study revealed that the absence of flourishing mental health was associated with increased all-cause mortality [15]. A randomized control trial showed that promoting flourishing mental health decreased anxiety and depressive symptoms [16].

In addition, a growing body of evidence has suggested the desirable correlates of flourishing and work-related outcomes. For example, flourishing employees demonstrate higher levels of productivity, put greater effort in their work, report fewer missed workdays and work-related injuries, and lower levels of health care costs [17]. In a study from New Zealand, flourishing mental health among employees predicted elevated levels of satisfaction with work–life balance and job satisfaction [18]. Among workers in South Africa, several studies suggested that flourishing in work was strongly related to person–environment fit, job satisfaction, work engagement, and organizational commitment, and negatively related to turnover intention [19–21]. These findings suggest the importance of focusing on flourishing mental health among workers, not only for individual health, but also for the organization for which they work. However, research on flourishing employees is still in its infancy and the literature is limited. Working characteristics including occupational stress affect worker’s performance and motivation [22]. Therefore, it is possible that occupational stress is also associated with flourishing mental health. However, to date, the association has not been fully elaborated. If occupational stress affects workers’ flourishing mental health, managers should try to change the factor that facilitates well-being to maximize work-related outcomes.

There are two reasons to worth conducting an epidemiological study on positive aspects of mental health among workers in Japan. First, research on employees’ mental health in terms of well-being is scarce. In Japan, the increasing incidence of mental illness and suicide among workers has received attention for decades, and the importance of prevention of them has gained widespread acceptance [23]. By contrast, little attention is paid to positive aspects of mental health. Therefore, mental health promotion in Japan remains limited to mental illness prevention initiatives such as reducing the amount of overtime work or introducing stress check systems [24]. The reason for the current dominance of mental illness over mental health in the Japanese occupational health research field may be explained by the significant lack of epidemiological evidence related to positve aspects of mental health in the workplace. Although the traditional focus on the occupational stress-related epidemiology of depression, anxiety, burnout, absenteeism, and other adverse health outcomes has produced important evidence, it has not provided a clear picture of well-being of the working population.

Secondly, flourishing mental health can play a key role in maintaining the workforce in Japan for its beneficial effect. In 2018, the labor force in Japan was approximately 74.8 million (59.8% of the population) and the number is declining year by year, resulting from an aging population and a falling birth rate. Considering the declining working population in Japan, the Japanese government has begun promoting the participation of women, the elderly, and people with disabilities in the workforce, including those raising small children and caring for ailing relatives to compensate for the labor shortage. It is therefore essential to cultivate flourishing mental health not only for individual health, but also for optimal functioning and productivity in the workforce**.**

To promote well-being and productivity in the workforce, knowledge about the prevalence and determinants of flourishing mental health is important. Such epidemiological information is also an essential component of a policy making and will contribute to the development of occupational research for workers’ well-being in Japan. Therefore, to test our hypothesis that flourishing mental health is associated with work characteristics, we conducted a cross-sectional, web-based survey focusing on flourishing mental health among workers in Tsukuba Science City, which is one of the largest research park cities in the world. Our aims were (1) to clarify the prevalence of flourishing mental health among Japanese workers and (2) to investigate the association between flourishing mental health and occupational stress, based on the demand–control model.

## Methods

### Study design and participants

This is a series of study using the dataset obtained in the 7th Living Condition and Workplace Stress Survey conducted by Tsukuba Science City Network (TSCN). The survey was a cross-sectional, web-based, and self-administered and aimed to investigate the mental health status, living conditions, and workplace environments of workers in Tsukuba Science City, Japan. A total of 53 organizational members of TSCN, which had a total of 19,481 workers, agreed to participate. Either Japanese version or English version of the questionnaire was available. Further information on the methodology used in the survey is available elsewhere [25, 26].

The variables analyzed in this study included mental health as measured by the Japanese version of the Mental Health Continuum–Short Form (MHC–SF), the Brief Scales for Job Stress (BSJS), sex, age group, marital status, educational attainment, annual household income (in Japanese yen), type of employment, occupation, physical activity, smoking status, and self-rated health, as well as numerous other items (the details of which are reported elsewhere) [25].

### The MHC–SF

The Japanese version of the MHC–SF, which is composed of 14 items, was used [11]. The first three items measure the degree of emotional well-being as defined in terms of happiness, satisfaction, and interest of life, respectively; the fourth through eighth items measure social well-being, with one item each on social acceptance, social actualization, social contribution, social coherence, and social integration, respectively; and the ninth through the fourteenth items measure psychological well-being, with one item each on autonomy, environmental mastery, personal growth, positive relations with others, purpose in life, and self-acceptance, respectively. Participants were asked to report on the following six-point scale how often they had felt the positive symptoms mentioned above over the past month: 0 = “never”, 1 = “once or twice”, 2 = “about once a week”, 3 = “about two or three times a week”, 4 = “almost every day”, and 5 = “every day.” According to Keyes et al. [12], flourishing mental health is defined as the coexistence of high hedonic well-being and high eudaimonic well-being. In those studies, participants were categorized as having high hedonic well-being if they scored 4 or 5 on at least one of three emotional well-being items, and as having high eudaimonic well-being if they scored 4 or 5 on at least six of eleven social and psychological well-being items. The MHC–SF and its subscales scores have shown high internal consistency and good discriminant validity with the hypothesized three-factor structure [27]. The MHC–SF is available in several languages and has shown excellent psychometrics and validities [12, 28–32]. The MHC-SF is translated in Japanese and used in other study [32]. However, the validity of the Japanese version has not been confirmed. Cronbach’s alpha coefficients in the present study were 0.921 for emotional well-being, 0.881 for social well-being, 0.906 for psychological well-being, and 0.949 for total MHC–SF score.

### Occupational stress

Chronic occupational stress perceived by the workers was assessed using the BSJS. The BSJS is a 20-item questionnaire developed by Nishikido et al. [33] based on the demand–control model, similar to the Job Content Questionnaire [34]. We asked participants to “select the response that most closely matches your feelings with regard to the descriptions about your current working circumstances”. Responses were given on a four-point rating scale (from 1 = “disagree” to 4 = “agree”), and the mean scores (range = 1.00–4.00) were calculated for six subscales (“workload,” “mental workload,” “problem in personal relationships,” “job control,” “reward from work,” and “support from colleagues and superiors”). For example, reward from work is measured by three items: “I am doing a job that I can take pride in and which is worthwhile,” “my job enables me to demonstrate my abilities,” and “I am doing a job that gives me a sense of accomplishment and satisfaction.” Workload, mental workload, and interpersonal relationships were defined as job-related stress, whereas job control, reward from work, and support from colleagues and superiors were defined as buffers against it. A higher score indicates a higher level of job-related stress or buffer against job-related stress. These subscales have shown sufficiently high internal consistency and validity in occupational health research [33, 35, 36]. The Cronbach’s alpha coefficients in the present study were 0.904 for workload, 0.855 for mental workload, 0.820 for interpersonal relationships, 0.864 for job control, 0.932 for reward from work, 0.845 for support from colleagues and superiors, and 0.806 for total BSJS score.

### Other variables

Age group was categorized into the following five groups: “20–29,” “30–39,” “40–49,” “50–59,” and “60–64” years. Respondents aged 65 years or older were excluded from the analysis because this is the age at which most of the workers in participating organizations retire. The respondents aged 65 years or older were likely reemployed for a special project or working in positions such as president or executive. Marital status was divided into three groups: “single,” “divorced/bereaved,” and “married.” Educational attainment was categorized into four groups: “high school,” “college, national institute of technology, or vocational school,” “university,” and “graduate school.” Annual household income (Japanese yen) was classified into four groups: < 4 million, 4–8 million, 8–12 million, and ≥ 12 million (as of December 2018, one million yen was equivalent to about 8800 US dollars or 7800 euro). Occupation was derived from the answer to the question: “Which of the following best describes your job?,” with the answer options as “researcher/academic,” “technician/engineer,” and “clerk/administration.” Type of employment was categorized into three groups: “part-time or temporary,” “full-time (fixed-term),” and “full-time (permanent).” Physical activity was measured with the questionnaire item “How often do you exercise for more than 30 minutes?” and classified into “less than once a month,” “Several times a month,” “once a week,” and “more than once a week.” Smoking habit was assessed with the question “Are you a current smoker?” and dichotomized into smoking and nonsmoking. Self-rated health was measured with the item “In general, how do you rate your health?,” with the answer options “very good,” “good,” “fair,” and “poor”; these answers were then dichotomized into “good/very good” and “fair/poor.”

### Statistical analysis

First, to compare proportions across the groups with and without flourishing mental health, we used the chi-squared test. In addition, we used the *t* test to compare the mean scores on the six scales of the BSJS. Second, to identify associations between flourishing mental health and job stress, we performed a series of binomial logistic regression analyses to calculate the adjusted odds ratios (AORs) and 95% confidence intervals (CIs). Flourishing mental health was used as the dependent variable, and six scales of the BSJS (continuous variables) were used as the independent variable. Model 1 included sex and age group, and model 2 was additionally adjusted for marital status, educational attainment, annual household income (yen), type of employment, occupation, physical activity, smoking status, and self-rated health. All statistical tests were two-tailed, and *p* values < 0.05 were regarded as indicating statistical significance. IBM SPSS 25 for Windows (IBM Corp., Armonk, NY) was used for all statistical analyses.

## Results

Of the 19,481 workers, 7255 completed the questionnaire (response rate, 37.2%). Participants aged 65 years and older and those who had missing values in investigated variables were excluded. Finally, data from 7012 participants were analyzed, with a valid response rate of 36.0% (7012/19481). The age distributions (mean ± standard deviation [SD]) of the participants were 44.0 ± 10.3 years for all participants (*n* = 7012), 45.0 ± 10.5 years for men (*n* = 4402), and 42.3 ± 9.6 years for women (*n* = 2610).

Table 1 summarizes the participants’ descriptive characteristics. The proportions of the participants classified as having flourishing mental health as measured by the MHC–SF was 12.4%. Among the participants, 37.2% were women, over 70% were married, nearly half had graduated from graduate school, and 15.1% had a household income of 12 million yen or above. In addition, 61.9% of the participants were employed full-time (permanent) and 41.6% were researchers/academics.

**Table 1:** Descriptive characteristics of the participants (*n* = 7012), Tsukuba, Japan (2017)

	Percent	Number
Mental health		
Flourishing	12.4	(870)
Non-flourishing	87.6	(6142)
Job-related stress, mean ± SD		
Workload	2.16	± 0.88
Mental workload	2.22	± 0.86
Problem in personal relationships	2.00	± 0.79
Buffer against job-related stress, mean ± SD		
Job control	2.76	± 0.76
Reward from work	2.77	± 0.86
Support from colleagues and superiors	2.80	± 0.69
Sex		
Men	62.8	(4402)
Women	37.2	(2610)
Age group		
20–29	9.3	(654)
30–39	25.9	(1818)
40–49	31.4	(2200)
50–59	26.9	(1885)
60–64	6.5	(455)
Marital status		
Never married	25.7	(1804)
Divorced/bereaved	3.8	(264)
Married	70.5	(4944)
Educational attainment		
High school	13.2	(923)
College, etc.	11.7	(823)
University	27.0	(1891)
Graduate school	48.1	(3375)
Annual household income (yen)		
Less than 4 million	15.4	(1082)
4–8 million	35.9	(2514)
8–12 million	33.6	(2359)
12 million or above	15.1	(1057)
Type of employment		
Part-time, temporary	20.7	(1448)
Full-time (fixed-term)	17.4	(1223)
Full-time (permanent)	61.9	(4341)
Occupation		
Researcher/academic	41.6	(2920)
Technician/engineer	22.5	(1578)
Clerk/administration	35.9	(2514)
Physical activity per week		
Less than once	53.3	(3735)
Once or more	46.7	(3277)
Smoking status		
Currently smoking	10.8	(757)
Not smoking	89.2	(6255)
Self-rated health		
Poor/fair	15.7	(1098)
Good/very good	84.3	(5914)

A comparison of characteristics between participants with flourishing and non-flourishing mental health is shown in Table 2. All variables investigated in the present study were statistically significantly associated with flourishing mental health. The scores for workload, mental workload, and problems in personal relationships were lower in the flourishing than in the non-flourishing participants. On the other hand, the scores for job control, reward from work, and support from colleagues and superiors were higher in the flourishing than in the non-flourishing participants. The participants with the following characteristics were more likely to have flourishing mental health compared with those without: aged 30–39 years, married, finished master’s or doctor’s degree, annual household income of more than 12 million yen, full-time staff (fixed-term), researcher/academic, exercise habit at least once a week, nonsmoking, and self-rated health as good/very good.

**Table 2:** Comparison of characteristics between flourishing and non-flourishing mental health (*n* = 7012), Tsukuba, Japan (2017)

	Flourishing		Non-flourishing		*p* value
	%	(*n*)	%	(*n*)	
Job-related stress, mean ± SD					
Workload	2.09	± 0.89	2.17	± 0.88	0.009
Mental workload	2.01	± 0.84	2.25	± 0.86	< 0.001
Problem in personal relationships	1.73	± 0.75	2.94	± 0.78	< 0.001
Buffer against job-related stress, mean ± SD					
Job control	3.11	± 0.78	2.71	± 0.75	< 0.001
Reward from work	3.36	± 0.77	2.69	± 0.84	< 0.001
Support from colleagues and superiors	3.20	± 0.68	2.72	± 0.67	< 0.001
Sex					0.003
Men	11.5	(506)	88.5	(3896)	
Women	13.9	(364)	86.1	(2246)	
Age group					< 0.001
20–29	13.1	(86)	86.9	(568)	
30–39	16.8	(305)	83.2	(1513)	
40–49	10.9	(240)	89.1	(1960)	
50–59	10.1	(191)	89.9	(1694)	
60–64	10.5	(48)	89.5	(407)	
Marital status					0.001
Never married	10.5	(190)	89.5	(1614)	
Divorced/bereaved	8.0	(21)	92.0	(243)	
Married	13.3	(659)	86.7	(4285)	
Educational attainment					< 0.001
High school	8.3	(77)	91.7	(846)	
College, etc.	9.1	(75)	90.9	(748)	
University	12.1	(228)	87.9	(1663)	
Graduate school	14.5	(490)	85.5	(2885)	
Annual household income (yen)					0.014
Less than 4 million	12.2	(132)	87.8	(950)	
4–8 million	10.8	(272)	89.2	(2242)	
8–12 million	13.6	(321)	86.4	(2038)	
12 million or above	13.7	(145)	86.3	(912)	
Type of employment					<0.001
Part-time, temporary	13.7	(199)	86.3	(1249)	
Full-time (fixed-term)	16.6	(203)	83.4	(1020)	
Full-time (permanent)	10.8	(468)	89.2	(3873)	
Occupation					<0.001
Researcher/academic	15.3	(448)	84.7	(2472)	
Technician/engineer	11.0	(174)	89.0	(1404)	
Clerk/administration	9.9	(248)	90.1	(2266)	
Physical activity per week					<0.001
Less than once	10.7	(399)	89.3	(3336)	
Once or more	14.4	(471)	85.6	(2806)	
Smoking status					0.020
Currently smoking	9.8	(74)	90.2	(683)	
Not smoking	12.7	(796)	87.3	(5459)	
Self-rated health					<0.001
Poor/fair	4.3	(47)	95.7	(1051)	
Good/very good	13.9	(823)	86.1	(5091)	

*SD* standard deviation

Statistical analyses were conducted with chi-squared test and *t* test

The results of binomial logistic regression analysis related to flourishing mental health are shown in Table 3. After adjusting for differences in sex and age, the results indicated statistically significant associations between flourishing mental health and workload, reward from work, and support from colleagues and superiors. On the contrary, mental workload was inversely associated with flourishing mental health. The statistically significant associations persisted after further controlling for marital status, educational attainment, annual household income, type of employment, occupation, physical activity, smoking status, and self-rated health. After adjusting for these factors, the results indicated statistically significant associations between flourishing mental health and workload (OR = 1.19, 95% CI = 1.05–1.36), mental workload (OR = 0.65, 95% CI = 0.57–0.75), reward from work (OR = 2.34, 95% CI = 2.04–2.68), and support from colleagues and superiors (OR = 1.67, 95% CI = 1.44–1.94).

**Table 3:** Binomial logistic regression analysis related to flourishing mental health (*n* = 7012), Tsukuba, Japan (2017)

	Sex and age group adjusted		Fully adjusted	
	AOR	95% CI^a^	AOR	95% CI^a^
Job-related stress (continuous)				
Workload	1.21	1.06–1.36	1.19	1.05–1.36
Mental workload	0.62	0.54–0.71	0.65	0.57–0.75
Problem in personal relationships	0.90	0.80–1.01	0.93	0.82–1.05
Buffer against job-related stress (continuous)				
Job control	1.09	0.97–1.24	1.08	0.95–1.23
Reward from work	2.43	2.14–2.77	2.34	2.04–2.68
Support from colleagues and superiors	1.68	1.45–1.94	1.67	1.44–1.94
Sex				
Men, reference	1.00		1.00	
Women	1.30	1.10–1.54	1.28	1.05–1.56
Age group				
20–29, reference	1.00		1.00	
30–39	1.24	0.94–1.64	1.05	0.78–1.43
40–49	0.85	0.64–1.12	0.71	0.52–0.98
50–59	0.82	0.61–1.10	0.67	0.47–0.94
60–64	0.82	0.54–1.22	0.56	0.36–0.87
Marital status				
Never married, reference			1.00	
Divorced/bereaved			0.97	0.58–1.63
Married			1.44	1.15–1.80
Educational attainment				
High school, reference			1.00	
College, etc.			0.87	0.61–1.25
University			1.19	0.88–1.60
Graduate school			1.19	0.87–1.64
Annual household income (yen)				
Less than 4 million, reference			1.00	
4–8 million			0.83	0.64–1.08
8–12 million			1.11	0.84–1.48
12 million or above			0.94	0.68–1.32
Type of employment				
Part-time, temporary, reference			1.00	
Full-time (fixed-term)			1.03	0.80–1.32
Full-time (permanent)			0.71	0.56–0.90
Occupation				
Researcher/academic, reference			1.00	
Technician/engineer			0.91	0.74–1.13
Clerk/administration			1.02	0.81–1.29
Physical activity per week				
Less than once			1.00	
Once or more			1.39	1.19–1.62
Smoking status				
Currently smoking, reference			1.00	
Not smoking			1.11	0.84–1.45
Self-rated health				
Poor/fair, reference			1.00	
Good/very good			1.88	1.37–2.59
Nagelkerke *R*^2^	0.19		0.21	

*OR* odds ratio, *CI* confidence interval

^a^95% CI is shown as “lower-bound–upper-bound”

## Discussion

The aim of this study was to estimate the prevalence of flourishing mental health and evaluate the association between flourishing mental health and occupational stress among workers in Japan. To the best of our knowledge, this is the first large-scale epidemiological study in Japan to describe the prevalence of flourishing mental health. Moreover, precedent epidemiological studies of flourishing mental health were mainly conducted among the general population. Therefore, our results advanced the existing literature by providing evidence on occupational settings.

### Prevalence of flourishing among workers

Our study revealed that 12.4% of workers had flourishing mental health. Because there is no epidemiological study regarding the prevalence of flourishing in Japan, we compared the result with that from the other countries. Our result showed smaller prevalence than that in Canada (76.9%) [10], in the Netherlands (36.5%) [14], in South Africa (20.0%) [12], or in the USA (18.1%) [11]. On the other hand, our result showed slightly greater prevalence than that in South Korean adults (11.7%) [13]. Few epidemiological studies have examined the prevalence of flourishing mental health among workers. One exception is Hone et al. [18] who reported that 25% of workers in New Zealand were flourishing. Our findings suggest that there is much room for improvement in flourishing mental health among Japanese workers, compared to people in other countries. However, the result should be interpreted with caution because the assessment of flourishing, culture, subject sampling, age range, and geography differed from study to study. A sevenfold difference in the prevalence of flourishing was found between the lowest and highest countries in general population, which suggests cultural factors play a role in determining the prevalence. Iwata et al. have indicated the suppression of expression of positive emotions by the Japanese [37, 38]. Another possible explanation for the low level of mental health in the current study is the higher level of national inequality and collectivism, which are known to be predictors of subjective well-being across countries [39]. However, it remains unknown whether these factors affect the prevalence of flourishing mental health among workers.

### Flourishing mental health and occupational stress

Flourishing workers appeared to enjoy fewer demanding jobs, less conflict with others, higher job control, higher rewards from work, and higher support compared with their non-flourishing peers. In terms of the demand–control model for occupational stress, the above results imply that flourishing workers tend to have a low strain job, which is defined as a job with low psychological demands and high job decision latitude. It is suggested that the risk of psychological strain and physical illness are lower in this condition compared with active jobs (high demands/high job decision latitude), high strain jobs (high demands/low job decision latitude), and passive jobs (low demands/low job decision latitude) [40]. This is in accordance with precedent studies suggesting a relationship between flourishing mental health and favorable health outcomes [15, 41]. It is possible that the characteristics of the participants in the present study affected the prevalence of flourishing mental health as the majority were scientific researchers, who have been reported to enjoy a high level of job decision latitude [40].

Reward from work and support from colleagues and superiors were statistically significantly associated with flourishing mental health in the fully adjusted logistic regression analysis; these associations with were in-line with a precedent study [42]. Among the BSJS subscale scores, reward from work had the most influence on flourishing mental health. The result implies that retaining a focus on workers’ sense of that their job is worth doing, sense of using their strengths, and sense of accomplishment play an important role in flourishing mental health. Although it is practically difficult to improve the work environment, such as raising wages or reducing sales quotas, it might be possible to improve workers’ flourishing mental health via positive relationships in workplace. Acknowledgements, recognition, respect, or gratitude paid by workers’ colleagues and superiors could promote a sense of support among workers, as well as a feeling that their work is rewarding. Encouraging such mutual acts in the workplace might serve as an effective intervention to increase flourishing mental health. For example, Page and Vella-Brodrick [43] developed the working for wellness program for employee, which consisted of sessions to learn applying their strengths, or optimizing relationships at and outside of work.

Our results also showed that flourishing mental health was inversely associated with mental workload. It is essential for managers to support subordinate who are facing troubles or help new employees to orient themselves toward their work so that they can reduce their mental workload. Moreover, our result indicated that reducing mental workload only while keeping appropriate workload could be more effective in terms of maintaining good mental health. This was an important finding because managers often try to reduce both workloads as a countermeasure against mental illness. Managers should take this into account by monitoring employees’ perceived workloads to maximize the mental health in workplace.

### Flourishing mental health and other variables

It is noteworthy that part-time and temporary workers were significantly more flourishing than full-time (permanent) workers, after controlling for differences in household income and other socioeconomic factors. It is plausible that part-time or temporary workers experience more flourishing than full-time (permanent) workers because they generally have more free time and less workload. However, because of the cross-sectional design of the present study, it might be premature to conclude from our findings that part-time or temporary employment is good for well-being, as it is unstable compared with full-time employment. If a person is underemployed, working below their full capacity, or working for fewer hours than desired, unstable employment has a negative effect on mental health [44, 45]. Kume et al. [46] pointed out that involuntary part-time employment is negatively associated with subjective happiness. Future studies should therefore address the issue of whether the participants’ present type of employment is voluntary or involuntary.

We identified a pattern of gradually decreasing numbers of flourishers with increasing age. This gradual pattern for age group is in accordance with a report in which adults in Japan aged 50–59 years reported the lowest level of happiness [47]. This finding is in contrast with Keyes and Simoes [15], who reported finding the highest rate of flourishers in the 45–54-year-old group in the USA. Thus, older workers in Japan might be a good target for promoting flourishing mental health. Female or married participants were more likely to have flourishing mental health. This finding was in agreement with other studies in terms of predictors of flourishing mental health [10, 15, 48]. On the contrary, the highest level of educational attainment and household income was not significantly associated with flourishing mental health after controlling for other factors. It is possible that high income and educational background are not requirements for flourishing mental health in Japanese workers. This result suggests that mental health promotion strategies in workplace should be aimed at enhancing social rewards and support instead of raising wages.

The result of the multinomial logistic regression analysis indicated that self-rated health plays an important role in relation to flourishing mental health. Although more than 80% of the participants rated their health as good or very good, only 12.4% were categorized as having flourishing mental health. Other studies have also identified a gap between flourishing mental and self-rated health [13, 49]. This result implies that perceived health is not a sufficient condition for flourishing mental health. It is therefore essential to pay attention not only to workers’ general health, but also to their positive aspects of mental health.

## Strengths and limitations

The present study had several strengths. To our knowledge, this is the first study to demonstrate the prevalence of flourishing mental health among Japanese workers. Moreover, we showed association between flourishing mental health and occupational stress. Previous epidemiological studies on flourishing mental health have been conducted mainly in Western countries and with general populations. In addition, our study had a considerable sample size, with a total of 7012 workers.

However, the present study also had some limitations. First, the validity of the Japanese version of the MHC-SF, main outcome of the current study, has not been confirmed in precedent studies. Future research is needed to establish validity and utility of the Japanese version of the MHC-SF. Second, because of the cross-sectional design, causal relationships could not be determined. The mechanisms underlying the association between flourishing mental health and job stress therefore remains unclear. Flourishing workers might be engaging in their work enthusiastically so that they can gain social rewards or support in the workplace, or vice versa. A longitudinal study is therefore required to disentangle the causal relationships between flourishing mental health and occupational stress. Third, the response rate was relatively low, so there may have been a non-response or other type of bias that could have affected the results. A number of participants, especially non-flourishing workers, might have been reluctant to respond to the questionnaire. Thus, our results regarding the prevalence of flourishing mental health might be overestimated. Future survey with high response rate is needed to clarify the accurate prevalence. Fourth, our target population was not a random sample from the general working population in Japan. Our participants included many researchers, highly educated and earning individuals. Moreover, Kageyama et al. revealed that scientific researchers had higher score of “reward from work” than other job [36]. Therefore, it is possible that the prevalence of flourishing might be overestimated in our study. Some caution is therefore required when generalizing the findings to workers in general. Fifth, not all factors related to flourishing mental health were considered in the current study. For example, medical history, drinking status, and objective work-related indicators, such as working hours, should also be considered in a future study. Lastly, all data were gathered from self-administered measures. Therefore, there is a possibility that a part of the observed significant associations may be attributed to common-method bias.

## Conclusion

Our study revealed that 12.4% of workers in Tsukuba Science City had flourishing mental health. This is the first large epidemiological study in Japan to demonstrate the prevalence of flourishing mental health. Moreover, our findings shed light on the association between occupational stress and flourishing mental health. These results are useful in developing positive mental health promotion strategies. Undoubtedly, further research on flourishing mental health in Japan is needed. The effect of flourishing mental health on illness, mortality, and other important public health outcomes had not yet elucidated in the East Asia.

## Data Availability

The data that support the findings of this study are available from TSCN but restrictions apply to the availability of these data, which were used under license for the current study, and so are not publicly available. Data are however available from the authors upon reasonable request and with permission of TSCN.

## Abbreviations

AOR: Adjusted odds ratio
BSJS: Brief Scales for Job Stress
MHC-SF: Mental Health Continuum Short Form
OR: Odds ratio
SD: Standard deviation
TSCN: Tsukuba Science City Network
